# Antithrombotic Therapy in Transcatheter Aortic Valve Implantation: Focus on Gender Differences

**DOI:** 10.3390/jcdd12110433

**Published:** 2025-11-02

**Authors:** Mattia De Gregorio, Andrea Denegri, Filippo Luca Gurgoglione, Giorgio Benatti, Iacopo Tadonio, Emilia Solinas, Davide Carino, Andrea Agostinelli, Luigi Vignali, Giampaolo Niccoli

**Affiliations:** 1 Division of Cardiology, Parma University Hospital, 43126 Parma, Italy; 2 Department of Medicine and Surgery, University of Parma, 43121 Parma, Italy; 3 Cardiac Surgery Unit, University Hospital of Parma, 43126 Parma, Italy

**Keywords:** TAVI, antithrombotic therapy, gender, women, outcomes

## Abstract

Antithrombotic therapy plays a pivotal role in reducing thromboembolic complications, including stroke and valve thrombosis, following Transcatheter Aortic Valve Implantation (TAVI). However, the benefits of such therapy must be balanced against the increased risk of major bleeding events. The optimal antithrombotic strategy in this setting remains a matter of ongoing debate, given the heterogeneity of patient profiles and procedural variables. Among TAVI recipients, women represent a growing proportion and exhibit distinct anatomical, physiological, and clinical characteristics that influence both thrombotic and bleeding risk. Compared to men, women more frequently experience vascular complications and major bleeding events, despite better survival outcomes. These differences are driven by smaller vessel caliber, higher vascular tortuosity, and altered platelet reactivity. Consequently, sex-specific risk stratification is essential when considering antiplatelet or anticoagulant regimens post-TAVI. This review provides a comprehensive synthesis of current evidence regarding antithrombotic strategies in the post-TAVI setting, with a dedicated focus on sex-related differences. Particular emphasis is placed on the female population, assessing ischemic and hemorrhagic outcomes and the implications for long-term management. Improving outcomes in women undergoing TAVI necessitates tailored antithrombotic strategies that balance efficacy and safety. Ongoing research and dedicated trials are essential to refine these strategies and to inform future guideline updates in this expanding patient population.

## 1. Introduction

Transcatheter Aortic Valve Implantation (TAVI) has revolutionized the management of severe aortic stenosis, particularly in elderly patients and those at elevated surgical risk [1]. As the procedure becomes increasingly utilized in intermediate- and low-risk populations, attention has shifted from procedural success to long-term outcomes, including thromboembolic and hemorrhagic complications [2,3]. Post-procedural antithrombotic therapy plays a central role in mitigating ischemic events such as stroke, myocardial infarction, and valve thrombosis [4]. However, these benefits must be weighed against the considerable risk of bleeding, especially in frail patients with multiple comorbidities [5].

One emerging area of interest is the role of biological sex in influencing outcomes following TAVI. Women often exhibit a unique profile of anatomical and physiological characteristics—such as smaller vascular caliber, increased vascular tortuosity, and differing hemostatic profiles—that impact both thrombotic and bleeding risks. Although women experience higher rates of vascular and bleeding complications, survival outcomes remain comparable or superior to those of men, highlighting the complex interplay between sex, procedural factors, and pharmacologic management [6].

This review aims to explore the rationale behind current antithrombotic strategies post-TAVI, analyze existing guideline recommendations, and evaluate sex-specific outcomes, with a particular focus on the female population. We also discuss the need for individualized therapeutic approaches and the implications for clinical decision-making in the evolving landscape of structural heart interventions.

## 2. Rationale for Antithrombotic Therapy

The rationale for antithrombotic therapy following TAVI arises from the inherent thrombogenicity of the prosthetic valve’s metallic scaffold, particularly during the early post-procedural period prior to complete reendothelialization [7]. Post-TAVI patients are exposed to a dynamic and elevated risk of thromboembolic events, including myocardial infarction (MI), valve thrombosis, transient ischemic attack (TIA), and ischemic stroke [8,9].

This risk is exacerbated by multiple patient-related factors, such as advanced age, a history of cerebrovascular events, left ventricular systolic dysfunction, atrial fibrillation, and additional comorbidities—including obesity, chronic kidney disease, and anemia—that may contribute to a prothrombotic milieu, which is particularly prevalent in the TAVI population [10,11,12,13,14]. Moreover, the risk of thrombosis is determined also by local factors such as the exposure of tissue factor due to disruption of the native aortic valve leaflets, altered flow dynamics, and areas of low shear stress resulting from the incomplete expansion or malposition of the prosthesis [15,16,17,18,19]. These conditions promote thrombus formation in the sinuses of Valsalva, particularly in patients with small sinuses, in valve-in-valve procedures, with the use of balloon-expandable devices or post-dilation, which has been associated with an increased incidence of cerebrovascular events [20,21,22,23,24,25]. Gender specific antithrombotic outcomes after TAVI are summarized in Table 1.

Clinically significant obstructive valve thrombosis is observed in approximately 0.5% of patients annually and is defined by a transvalvular gradient increase ≥10 mmHg from baseline or an absolute mean gradient > 20 mmHg [26]. This may present with recurrent symptoms of aortic stenosis, new-onset heart failure, or thromboembolic complications. In contrast, subclinical leaflet thrombosis, typically detected via four-dimensional computed tomography (4D-CT) imaging, is more common—affecting 7% to 15% of TAVI recipients—and is characterized by hypoattenuated leaflet thickening (HALT) with or without reduced leaflet motion (RLM) [27,28]. Together, these findings underscore the critical importance of tailored antithrombotic strategies to mitigate the heightened thromboembolic risk following TAVI, particularly during the vulnerable early post-procedural phase.

## 3. Bleeding Risk Assessment

While antithrombotic therapy remains essential to prevent thromboembolic complications following TAVI, it significantly increases the risk of bleeding—particularly in elderly, frail patients with multiple comorbidities. Several clinical and anatomical factors contribute to post-procedural hemorrhagic complications in this population.

Advanced age, renal or hepatic dysfunction, frailty, and acquired thrombocytopenia are established predictors of bleeding events [29]. Notably, thrombocytopenia may be aggravated by mechanical shear stress at the site of the implanted valve, leading to platelet destruction. Moreover, aortic stenosis is frequently associated with gastrointestinal arteriovenous malformations and acquired von Willebrand factor deficiency, both of which predispose to mucosal and gastrointestinal bleeding [30,31]. Concomitant coronary artery disease and atrial fibrillation are common in TAVI candidates and necessitate the use of antiplatelet or oral anticoagulant therapies, thereby compounding the bleeding risk. In addition, the percutaneous nature of the procedure, involving large-bore arterial access, increases the likelihood of vascular complications, especially in patients with peripheral arterial disease. Importantly, most TAVI studies have been conducted in elderly cohorts with overlapping risk factors for both thrombosis and bleeding. As TAVI is increasingly used in younger and lower-risk patients, the benefit–risk profile of antithrombotic strategies is expected to shift. Thus, individualized risk stratification becomes essential in balancing ischemic protection with bleeding avoidance.

Future research should aim to refine risk prediction models and identify biomarkers to guide the selection and duration of antithrombotic therapy in diverse patient subgroups, including those at high bleeding risk.

## 4. Sex-Specific Outcomes in Women Undergoing TAVI

Sex-based differences in clinical outcomes following TAVI have garnered increasing attention. Several large-scale meta-analyses have demonstrated that women undergoing TAVI are at higher risk for major bleeding, vascular complications, and stroke compared to men [32,33]. However, these adverse events do not appear to translate into higher mortality. Indeed, survival rates between sexes are largely comparable.

In a pooled analysis by Viastra et al., 30-day mortality was similar between women and men (5.9% vs. 5.5%, respectively; *p* = 0.17), with no significant difference in stroke incidence (2.3% vs. 2.5%; *p* = 0.53). However, women exhibited a 50% increased risk of life-threatening or major bleeding compared to men (6.7% vs. 4.4%; *p* < 0.01) [34].

**Figure 1:**
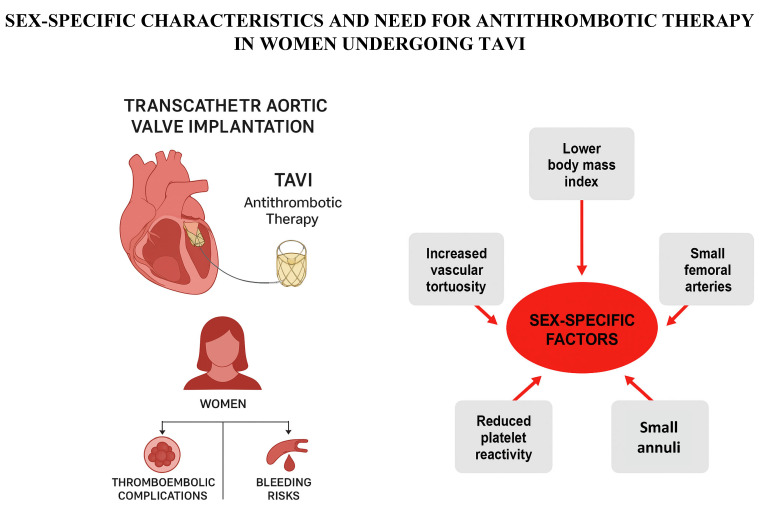
Sex-specific factors that may influence outcome after TAVI in women and determine the need for anti-thrombotic treatment.

These discrepancies are likely attributable to anatomical and procedural factors (Figure 1). Women typically present with smaller body size, lower body mass index (BMI), and smaller iliofemoral arteries, leading to higher sheath-to-artery ratios and increased vascular tortuosity—factors known to elevate the risk of access-site complications [35,36]. The bleeding risk is particularly pronounced in women with low BMI [34]. In addition, sex-related differences in platelet function have been observed. Women often exhibit prolonged bleeding times and reduced platelet reactivity compared to men, further predisposing them to hemorrhagic events [37].

The RHEIA trial—the first randomized study dedicated exclusively to women undergoing aortic valve replacement—demonstrated that TAVI is not only non-inferior but potentially superior to surgical aortic valve replacement (SAVR) in terms of composite clinical outcomes. In this multicenter trial enrolling 443 women with severe symptomatic aortic stenosis, the composite endpoint of all-cause mortality, stroke, or heart failure rehospitalization at one year was significantly lower in the TAVI group (8.9%) compared to the SAVR group (15.6%; HR 0.55; 95% CI 0.34–0.88; *p* = 0.03). This benefit was primarily driven by a reduction in valve-related or heart failure–related rehospitalizations [38].

These findings emphasize the need for sex-specific risk assessment and highlight the importance of individualized procedural planning and antithrombotic management in female patients undergoing TAVI.

## 5. Current Guideline Recommendations

Optimal antithrombotic therapy following TAVI requires a careful balance between thromboembolic protection and bleeding risk mitigation. Current international guidelines provide recommendations that are primarily stratified based on the presence or absence of an indication for long-term oral anticoagulation (OAC).

The 2021 ESC/EACTS guidelines recommend lifelong single antiplatelet therapy (SAPT)—typically aspirin 75–100 mg/day or clopidogrel 75 mg/day—for patients undergoing TAVI without a clear indication for OAC (Class I, Level of Evidence A). In patients who do require OAC, the use of long-term OAC alone (either a vitamin K antagonist [VKA] or direct oral anticoagulant [DOAC]) is recommended without the addition of antiplatelet agents (Class I, Level of Evidence B). For patients with recent coronary stent placement, dual antiplatelet therapy (DAPT)—aspirin plus clopidogrel—is advised, with duration determined by the individual’s bleeding risk. Typically, DAPT is administered for 1 to 6 months, followed by lifelong SAPT [39].

The 2020 ACC/AHA guidelines similarly support SAPT in patients without an indication for OAC and suggest VKA therapy (target INR ~2.5) for at least 3 months post-TAVI in patients with low bleeding risk who require anticoagulation [40]. In patients with conditions such as atrial fibrillation, venous thromboembolism, or hypercoagulable states, continuation of OAC post-TAVI is mandatory. Both VKAs and DOACs are acceptable options, with therapy tailored to individual clinical profiles.

A 2021 Delphi consensus statement from experts in the United Kingdom and Ireland emphasizes that the duration and type of antithrombotic therapy post-TAVI should be individualized rather than universally applied [41]. For high-bleeding-risk patients, SAPT duration may range from 3 to 12 months, with shorter regimens preferred. However, in the presence of concomitant coronary artery disease, longer SAPT or temporary DAPT may be necessary.

Additionally, the 2021 ESC/EAPCI expert consensus document offers further recommendations for patients undergoing TAVI with recent percutaneous coronary intervention (PCI). For those without an indication for OAC, DAPT (aspirin plus clopidogrel) should be considered for 1 to 6 months, depending on the indication (1 to 3 months for chronic coronary syndrome (CCS) and 3 to 6 months for acute coronary syndrome (ACS). Subsequently, therapy should transition to lifelong SAPT [42,43].

These evolving recommendations underscore the importance of personalized treatment strategies, considering ischemic and bleeding risks, comorbidities, and procedural factors.

## 6. Emerging Evidence from Randomized Clinical Trials

While current guidelines provide foundational recommendations for antithrombotic therapy post-TAVI, clinical practice continues to evolve based on emerging data from randomized controlled trials (RCTs). These trials explore the comparative safety and efficacy of various antiplatelet and anticoagulant regimens, particularly in patients with and without indications for long-term oral anticoagulation. Below, we summarize key findings from pivotal RCTs that have informed and challenged existing clinical paradigms.

### 6.1. Antiplatelet Therapy

Several open-label, randomized controlled trials have evaluated the efficacy and safety of different antiplatelet strategies in patients undergoing TAVI without a pre-existing indication for OAC.

The ARTE trial (2017) was the first to compare dual antiplatelet therapy (DAPT: aspirin + clopidogrel) with single antiplatelet therapy (SAPT: aspirin alone). At 90-day follow-up, the composite endpoint of death, myocardial infarction (MI), stroke, transient ischemic attack (TIA), or major/life-threatening bleeding occurred in 15.3% of DAPT patients versus 7.2% in the SAPT group (*p* = 0.065). Major or life-threatening bleeding was significantly higher in the DAPT group (10.8% vs. 3.6%, *p* = 0.038), with no available sex-stratified results [44].

The POPular TAVI Cohort A trial randomized patients to aspirin monotherapy or aspirin plus clopidogrel for three months post-TAVI. At 12 months, overall bleeding occurred in 15.1% of the SAPT group versus 26.6% in the DAPT group (RR 0.57; 95% CI: 0.42–0.77; *p* = 0.001). Non-procedural bleeding was also lower in SAPT (15.1% vs. 24.9%, RR 0.61; *p* = 0.005) [45].

The OCEAN-TAVI registry explored an unconventional approach, comparing SAPT, DAPT, and no antithrombotic therapy post-TAVR. Notably, the no-antithrombotic group did not demonstrate a higher incidence of cardiovascular death, stroke, or major bleeding, and valve performance remained similar across all groups [46]. However, this study was retrospective in design, and randomized trials are needed to confirm whether selected patients might benefit from complete antithrombotic avoidance.

Collectively, these findings suggest that single antiplatelet therapy may offer a more favorable safety profile than dual therapy in TAVI patients, although further randomized data—particularly with sex-stratified analyses—are warranted to refine patient-specific antithrombotic strategies.

### 6.2. Anticoagulant Therapy

#### 6.2.1. Patients Without a Prior Indication for OAC

The detection of subclinical leaflet thrombosis (SLT) via four-dimensional computed tomography (4D-CT) raised concerns about potential thromboembolic risk in TAVI patients without an existing indication for OAC [27]. Contributing anatomical and procedural factors include small sinuses of Valsalva, low coronary washout, intra-annular valve positioning, valve-in-valve procedures, and commissural misalignment [47,48,49].

Although the clinical relevance of SLT remains debated, some studies suggest an association with increased risk of cerebrovascular events (RR ~ 2.6), while others report no significant impact on clinical events [50,51,52,53,54].

The GALILEO trial (n = 1644) compared rivaroxaban 10 mg daily (plus aspirin for 3 months) versus DAPT followed by aspirin alone. The trial was terminated early due to higher mortality, bleeding, and thromboembolic complications in the rivaroxaban group [55]. Women (n = 813) were older and had a higher STS compared to men (n = 831); nevertheless, women presented with a lower risk of MACE (HR 0.69), all-cause mortality (HR 0.54), and non-cardiovascular mortality (HR 0.33) and no significant difference in major bleeding, CV death, or stroke [56].

The ATLANTIS trial evaluated apixaban (5 mg BID) versus standard antithrombotic therapy. In patients without OAC indication (Stratum 2, n = 1049), apixaban reduced SLT incidence but did not significantly improve composite thromboembolic or bleeding outcomes and was associated with more non-cardiovascular deaths [57]. Similarly, the ADAPT-TAVR trial confirmed that DOAC therapy reduces SLT compared to DAPT, but this did not translate into fewer strokes or cardiovascular events [58,59]. Neither ATLANTIS nor ADAPT-TAVR has reported sex-stratified results.

#### 6.2.2. Patients with a Prior Indication for OAC

The POPular TAVI Cohort B trial assessed patients already on OAC (DOAC or VKA) who were randomized to receive OAC alone or OAC plus clopidogrel for 3 months. Bleeding complications, primarily at the vascular access site, were significantly lower in the OAC-only group, with no difference in ischemic outcomes [60]. Of note, no significant difference in total bleeding or ischemic events (MI, stroke) was detected (bleeding 22.8% vs. 23.6%, *p* = 0.815; ischemic events 5.6% vs. 7.0%, *p* = 0.429); however major or life-threatening bleeding was significantly higher in women compared to men (12.5% vs. 7.4%, *p* = 0.011), mostly driven by vascular access complications. In this group of patients, aspirin increased the risk of major bleeding in women but not in men [61].

The ENVISAGE-TAVI AF trial included 1426 patients with atrial fibrillation and compared edoxaban [factor Xa (FXa) inhibitor] with VKAs. Edoxaban was non-inferior for the composite outcome of death, MI, stroke, valve thrombosis, or major bleeding but was associated with a higher rate of gastrointestinal bleeding [62]. The overall treatment effect of edoxaban vs. VKA appears broadly consistent across sexes, with a slightly attenuated bleeding risk in women (n = 658, 47.8%), albeit without statistical confirmation.

A meta-analysis of eight studies, including both RCTs and observational cohorts [63,64,65,66,67,68,69], found no difference in all-cause mortality, stroke, or major bleeding between DOACs and VKAs in TAVR patients with AF. However, the risk of overall bleeding was lower in patients receiving DOACs.

These randomized trials provide critical insights into optimizing antithrombotic therapy after TAVR. While SAPT appears safer than DAPT in most patients without OAC indication, the role of DOACs versus VKAs in patients with established anticoagulation needs remains under active investigation. These findings are summarized in Table 2.

### 6.3. Antiplatelet, Anticoagulation, or Antithrombotic?

As TAVI continues to expand into lower-risk and younger populations, optimizing antithrombotic strategies remains a dynamic and evolving challenge. Future research should focus on identifying patient-specific biomarkers and imaging modalities that can predict thrombotic and bleeding risks with higher precision, allowing for a more tailored approach to therapy.

Randomized trials powered for sex-specific endpoints are particularly needed to address the unique ischemic-hemorrhagic profile observed in women undergoing TAVI. Furthermore, ongoing trials evaluating ultra-short DAPT durations, aspirin-free regimens, and the role of direct oral anticoagulants (DOACs) in various risk strata will inform future guideline updates. The emerging field of precision antithrombotic therapy, incorporating genetic testing, pharmacogenomics, and artificial intelligence-based risk stratification tools, holds promise for advancing individualized care.

Another unmet need lies in long-term monitoring of subclinical leaflet thrombosis (SLT), which is increasingly recognized as a potential driver of cerebrovascular events and valve dysfunction. Standardized imaging protocols and longitudinal follow-up studies will be essential to elucidate the clinical consequences of SLT and determine the most effective preventive strategies.

Finally, integrating sex-based anatomical and physiological differences into device design and procedural planning may reduce vascular and bleeding complications, particularly in women. A correct assessment of the SFAR (Sheath-to-Femoral Artery Ratio) at CTA, with high risk defined as values > 1.05, allows possible vascular complications to be predicted. In high-risk populations, it is therefore necessary to carefully select the puncture site using the necessary equipment (primarily by performing ultrasound-guided puncture), evaluating alternative sheaths, alternative accesses (especially for SFAR > 1.20) such as apical, axillary, or carotid access (in experienced centers), dilations with progressive caliber introducers, use of protamine at the end of the procedure (if not contraindicated), and use of hybrid access closure systems (suture-based + plug-based). As we move toward a more patient-centered and risk-adapted treatment paradigm, future guidelines must reflect this complexity by supporting shared decision-making and individualized antithrombotic therapy.

## 7. Hormonal Influence and Sex-Related Variability

Sex hormones—particularly estrogens and progesterone—significantly influence coagulation, platelet function, and fibrinolysis, thereby modulating the efficacy and safety of antithrombotic therapy in women. Postmenopausal estrogen decline promotes a prothrombotic state, characterized by increased fibrinogen, reduced antithrombin III and protein C activity, and elevated plasminogen activator inhibitor-1 (PAI-1), while hormone replacement therapy (HRT) only partially reverses these changes [70,71,72,73]. Estrogen therapy also enhances platelet activation and fibrin turnover, as evidenced by increased P-selectin and D-dimer levels [71]. Clinically, these hormonal shifts may alter bleeding and thrombotic risk, as seen in the higher incidence of heavy menstrual bleeding among premenopausal women receiving direct oral anticoagulants compared with vitamin K antagonists [74,75]. Therefore, stratifying TAVI patients by menopausal status, endogenous estrogen levels, and HRT use is essential to optimize antithrombotic strategies and accurately interpret sex-specific outcomes.

## 8. Future Perspectives

The findings from recent clinical trials and registries underscore the complexity of managing antithrombotic therapy in TAVI recipients, particularly among women. Despite improved valve technologies and procedural techniques, women continue to experience disproportionately higher rates of bleeding and vascular complications. These disparities can be attributed to smaller iliofemoral arteries, higher sheath-to-artery ratios, and sex-specific differences in platelet reactivity and endothelial function.

The increased bleeding risk in women does not appear to compromise survival outcomes; rather, some studies suggest more favorable mortality trends in females compared to males post-TAVI. This paradox—of higher procedural complication rates yet similar or superior survival—highlights the resilience of female physiology and the potential influence of factors such as smaller left ventricular mass and better left ventricular recovery.

Guidelines from ESC, ACC/AHA, and expert consensus statements advocate for SAPT in patients without indications for oral anticoagulation and recommend tailored approaches based on ischemic and bleeding risk profiles. However, these guidelines are largely based on data derived from predominantly male or mixed populations, with limited sex-specific stratification.

The RHEIA trial represents a major step forward in generating evidence exclusively from female cohorts and supports the safety and efficacy of TAVI in women. Nevertheless, further randomized trials are needed to validate sex-specific antithrombotic protocols and clarify optimal therapeutic durations. A randomized trial (NCT05493657) is underway to evaluate single therapy with clopidogrel vs. ASA, but the results are not yet available. When available, these data could be particularly useful in patients at higher risk of bleeding, such as in the female population.

A precision medicine approach—integrating clinical risk scores, imaging data, and biomarkers—may help guide antithrombotic decisions and improve outcomes, particularly in subgroups at the extremes of risk.

## 9. Conclusions

Antithrombotic therapy following TAVI is essential for preventing thromboembolic complications but carries a significant bleeding risk, particularly in women. Female patients present a distinct risk-benefit profile due to unique anatomical and physiological characteristics that must be considered when selecting antithrombotic regimens. Moving forward, sex-specific research and evidence-based protocols will be essential to improve safety and efficacy in this expanding patient population. A nuanced understanding of gender-related differences should inform both clinical practice and future guideline development in structural heart interventions.

## Figures and Tables

**Table 1:** Summary of Observational Registries Reporting Gender-Specific Antithrombotic Outcomes After TAV.

Study	Population	Treatment Comparison	Findings
WIN-TAVI	1000 women	DAPT vs. SAPT vs. OAC	At 1 year, most patients are on SAPT or no therapy. No male comparator. High bleeding risk in elderly females. Following multivariable stepwise Cox regression, DAPT use results as a borderline independent predictor of the 1-year primary VARC-2 efficacy endpoint (HR: 0.70; 95% CI: 0.49 to 1.01; *p* = 0.059).
FRANCE-TAVI	>10,000 (mixed)	SAPT/OAC/DAPT	Female sex is associated with a higher risk of major bleeding and vascular complications. Male sex independently predicted mortality (HR: 1.63; 95% confidence interval [CI]: 1.44 to 1.84; *p* < 0.001). Anticoagulation at discharge results is independently associated with lower rates of bioprosthetic valve dysfunction (OR: 0.54; 95% CI: 0.35 to 0.82; *p* = 0.005) but independently correlated with all-cause mortality (HR: 1.18 95% CI: 1.04 to 1.29; *p* = 0.013).
Eurointervention Registry	~1300 (mixed)	Per center protocol (heterogeneous)	Similar NACE in women (HR 1.16) and men (HR 1.08); edoxaban ↑ major bleeding in both but increase attenuated in women (HR 1.11 vs. 1.75 in men; interaction *p* = 0.170)
PARTNER 2 and 3 subanalysis	>1000	DAPT vs. SAPT (historical context)	Women had a higher risk of vascular complications (17.3% vs. 10.0%; 95% CI 4.63–9.95; *p* < 0.001) and major bleeding (10.5% vs. 7.7%; 95% CI 0.57–5.04; *p* = 0.012). Not primarily focused on therapy but supports anatomical susceptibility.

↑: increase.

**Table 2:** Randomized clinical trial on TAVI and anti-thrombotic treatment: gender differences (women vs. men).

Study	Year	Population/Women (%)	Treatment Comparison	Follow-Up	Findings
POPULAR TAVI	2020	978/46.7% women	Aspirin (±OAC) vs. DAPT	12 months	Total bleeding and ischemia similar; major/life-threatening bleeding higher in women (12.5 % vs. 7.4%; *p* = 0.011), especially with aspirin pre- and post-TAVI. In the SAPT vs. DAPT comparison, SAPT results in fewer bleeding events (15.1% vs. 26.6%; RR 0.57; 95% CI 0.42–0.77; *p* = 0.001). In OAC alone vs. OAC + antiplatelet, OAC alone results in fewer bleeding events (21.7% vs. 34.6%; RR 0.63; 95% CI 0.43–0.90; *p* = 0.011). Both strategies have non-inferiority for the composite ischemic endpoint.
ARTE	2017	222/36.9% women	3 mo. DAPT vs. ASA	3 months	No sex-stratified analysis available (overall trend: higher combined endpoint with DAPT, *p* = 0.065)
ENVISAGE TAVI AF	2021	1426/~47.5% women	Edoxaban vs. VKA (±antiplatelet)	540 days	Similar NACE in women (HR 1.16) and men (HR 1.08); edoxaban ↑ major bleeding in both; the relative increase is smaller in women than in men (HR 1.11 in women vs. 1.75 in men; interaction *p* = 0.170)
GALILEO	2020	~1644/49.5% women	Rivaroxaban + aspirin vs. DAPT	17 months	Women had a lower risk of MACE (HR 0.69), all-cause mortality (HR 0.54), and non-cardiovascular mortality (HR 0.33).
ATLANTIS	2022	~1510/53% women	Apixaban vs. standard care (antiplatelet or VKA)	12 months	No sex-stratified analysis available
ADAPT TAVR	2022	229 women (% not reported)	Edoxaban vs. DAPT	6 months	No sex-stratified data available

↑: increase.
